# Molecular correlates of swarming behaviour in *Aedes aegypti* males

**DOI:** 10.1098/rsbl.2024.0245

**Published:** 2024-10-30

**Authors:** Julien Devilliers, Hollie Marshall, Ben Warren, Charalambos P. Kyriacou, Luciana O. Araripe, Rafaela V. Bruno, Ezio Rosato, Roberto Feuda

**Affiliations:** ^1^ Neurogenetics Group,University of Leicester, Leicester, UK; ^2^ Department of Genetics,Genomics & Cancer Sciences, College of Life Sciences, University of Leicester, Leicester, UK; ^3^ Laboratório de Biologia Molecular de Insetos, Instituto Oswaldo Cruz (Fiocruz), Rio de Janeiro, Brazil

**Keywords:** *Aedes aegypti*, swarming, transcriptomics, circadian clock

## Abstract

Mosquitoes are the deadliest vectors of diseases. They impose a huge health burden on human populations spreading parasites as disparate as protozoans (malaria), viruses (yellow fever and more) and nematodes (filariasis) that cause life-threatening conditions. In recent years, mating has been proposed as a putative target for population control. Mosquitoes mate mid-air, in swarms initiated by males and triggered by a combination of internal and external stimuli. As the number of females in a swarm is limited, there is intense competition among males, and they ‘retune’ their physiology for this demanding behaviour. There is limited knowledge on the ‘genetic reprogramming’ required to enable swarming. Interestingly, recent evidence indicates that the upregulation of circadian clock genes may be involved in the swarming of malaria mosquitoes of the genus *Anopheles*. Here, we use whole-head RNA-seq to identify gene expression changes in *Aedes aegypti* males that are engaged in swarming in a laboratory setting. Our results suggest that in preparation to swarming, males tend to lower some housekeeping functions while increasing remodelling of the cytoskeleton and neuronal connectivity; the transcription of circadian clock genes is unaffected.

## 1. Introduction

Mosquitoes are important insect vectors as they transmit the microorganisms that cause malaria, filariasis and many viral diseases [1]. In the past century, the main strategies for population control have relied on insecticides and targeted haematophagous females. Currently, widespread resistance to pesticides and increased environmental concerns are urging the adoption of new approaches [2]. One promising strategy advocates using molecular tools to target key genetic regulators of development or reproduction [3,4]. With this approach, genetically modified males (non-biting) are mass-reared and released into the environment, where mating with resident females causes the local population to crash [3]. The underlying principle is that such males would compete effectively with their wild conspecifics to gain multiple access to females, which, depending on the species, tend to resist re-mating [5,6]. Swarming is the gathering of a large group of males flying together to attract females and competing for copulation. In mosquitoes, rivalry is particularly fierce. For instance, in *Anopheles gambiae* only *ca* 15% of males form copula pairs [7], while swarming depletes about 50% of their sugar and glycogen reserves [8]. Swarming is initiated by males and is prompted by a combination of internal and external stimuli, such as sex drive, visual landmarks, light levels, time of day, acoustic signals and olfactory cues [9]. However, the relative importance of these stimuli for swarming is still debated [10–15]. Additionally, we know little about how males ‘tune’ their physiology in preparation for this fundamental behaviour and the genetic regulations behind it. Such knowledge could provide clues for identifying the initial triggers of swarm formation and suggest measures to evaluate the fitness of mass-reared males before release.

We investigated changes in gene expression in swarming males of *Aedes aegypti*, a species with a wide geographical distribution and notable for spreading arboviruses that cause diseases such as dengue, Zika, chikungunya and urban yellow fever [1]. *Ae. aegypti* are diurnal mosquitoes, and swarming can take place at any time of day, although it is more common towards dusk if a host is not available [16]. A swarm forms when tens of males start flying in an erratic figure eight pattern around a host that provides olfactory cues [17]. Acoustic stimuli are also important, in that the acoustic signal generated by a male beating his wings in preparation for swarming may prompt nearby males to join [18]. Males aggregate and their pheromones attract females to enter the swarm [14]. During flight, females are recognized and chased based on their flight tone, which has a lower frequency compared to that of males [18]. Crucially, at the time of swarming, males increase the frequency of their flight tone, reaching approximately 1.5 times that of females, which is optimal for identifying them within the swarm [18,19].

To reveal transcriptional changes connected to swarming in *Ae. aegypti*, we performed mRNA-sequencing (RNA-seq) on whole heads of captive swarming and non-swarming males. We identified 27 differentially expressed transcripts; 11 were upregulated and 16 downregulated in swarming individuals. These changes correspond to increased expression of genes implicated in the remodelling of the cytoskeleton and neuronal connectivity, and reduced expression of those involved in some housekeeping functions.

In previous work, Wang *et al.* [20] investigated gene expression changes between swarming and non-swarming *Anopheles* males. They observed the core clock genes *period* (*per*) and *timeless* (*tim*) among the differentially expressed genes. Our work has not identified any overlap in the differentially expressed genes between the two species, including *per* and *tim*. However, we have observed upregulation of *slowpoke*, a gene previously implicated in the synchronization of circadian rhythmicity among neuronal clusters [21], and of the genes encoding the receptors *5-HT2A* and *Eph*, involved in the regulation of sleep and circadian behaviour [22,23]. Our results suggest that in *Ae. aegypti,* the expression of clock genes does not change at the transcriptional level around the time of swarming, at least in laboratory conditions. Yet, the changes that lead to sustained swarming behaviour possibly involve clock-relevant neuronal circuits.

## 2. Material and methods

### (a) Mosquitoes rearing

Animals were reared at the Laboratório de Biologia Molecular de Insetos, Instituto Oswaldo Cruz (Fiocruz, Rio de Janeiro, Brazil), under 12 h light–12 h dark (LD 12 : 12) conditions at 25°C. Pupae of *Ae. aegypti* (Rockefeller lineage) were provided by the Laboratório de Biologia, Controle e Vigilância de Insetos Vetores, IOC, Fiocruz. Pupae were maintained in plastic cups filled with water inside an experimental cage (metal frame of 40 × 40 × 40 cm with screens on all sides). After emergence, adults were fed with a 10% sucrose solution provided ad libitum inside the cage. The majority of pupae were males, but a few adult females (not sampled) were latter observed inside the cages and were left *in situ* during the experiment. Thus, not all males might have been virgin at the time of collection. However, as *Ae. aegypti* males readily mate multiple times [24], the females were very few, and as each cage contained more males than we collected, it is unlikely that mating status was a systematic criterion distinguishing swarming and non-swarming individuals.

### (b) Sampling and RNA extraction

We define swarming males those that aggregated in sustained flight in small groups in the centre of the cage. The proximity of the host (always the same researcher) was an extra stimulus for the males to engage in the behaviour. Irresponsive males standing on the cage walls were considered non-swarming. On average, the collection was completed within 10 min after the researcher entered the rearing room. Four independent samples were collected (17, 24 and 31 May and 7 June 2021), each consisting of 25 swarming and 25 non-swarming males from the same cage. In total, we collected 100 individuals per condition on the 4^th^ day post-emergence, at ZT10.5–11 (zeitgeber time, the time of the *time-giver*, is defined by the light switch, with ZT0 being lights on and ZT12 lights off). This sampling time corresponds to the activity peak in *Ae. aegypti* males [25]. Males were captured by aspiration and snap-frozen in liquid nitrogen. Heads were separated from bodies on ice and stored in TRIzol (Invitrogen) at −80°C until RNA extraction. Mosquito heads were homogenized in TRIzol with a motor-operated plastic pestle while thawing. The aqueous phase was isolated with the help of Phasemaker™ tubes (Thermo Fisher Scientific), and the RNA was precipitated with isopropanol using glycogen (Roche) as a carrier. A DNA-free kit (Invitrogen) was used to remove any contaminant genomic DNA. Sample quality was assessed using an Agilent RNA 6000 Nano Kit on an Agilent BioAnalyzer 2100 (Agilent Technologies), and a Qubit RNA BR Assay Kit on a Qubit 2.0 Fluorometer (Invitrogen). The eight samples were sequenced by NovoGene (UK) on a NovaSeq 6000 using 150 paired-end technology. The coverage was > 65 million reads per sample.

### (c) Reads mapping, differential gene expression and gene ontology enrichment analysis

We used FastQC 0.11.9 [26] to evaluate read quality and MultiQC 1.12 [27] to summarize results. Reads were aligned to the *Ae. aegypti* reference transcriptome (AeaegyptiLVP_AGWG, v. 5.7) from VectorBase [28], and their counts were extracted using Kallisto 0.44.0 [29]. We identified differential expression between swarming and non-swarming males with DESeq2 v. 1.40.2 [30] implemented in R v. 4.3.0 [31]. Transcripts with at least 10 counts were filtered, and samples were normalized by library size with the rlog transformation in DESeq2. We estimated differential transcript expression using a generalized linear model that considers data dispersion between samples. We corrected *p-*values according to the Benjamini–Hochberg method [32]. For the differentially expressed genes, a gene ontology enrichment analysis was performed with g:Profiler [33], using the expressed genes in the transcriptome as the background. We used VectorBase [28] and OrthoDB [34] to identify orthologues in *Drosophila melanogaster*, and FlyBase release FB2024_01 [35] to find information on gene function.

## 3. Results

**Figure 1.**
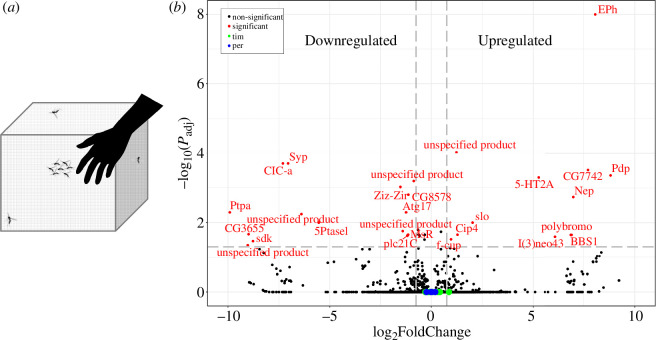
(*a*) Experimental design. Swarming males (flying) are attracted to a human host. Non-swarming males are irresponsive to the host and rest on the walls of the cage. (*b*) Volcano plot showing transcripts differentially expressed in swarming males (in red) (*p* < 0.05, log_2_FC > |0.75|). Transcripts of the circadian genes *period* (*per*) and *timeless* (*tim*) and are not differentially expressed and are shown in blue and green, respectively.

We performed whole-head (that includes the sensory structures for host seeking, swarming and mating) RNA-seq to identify genes that may underly physiological changes sustaining swarming in *Ae. aegypti*. We sampled swarming and non-swarming males towards the end of the day (ZT10.5–11.00, in a LD 12 : 12 regime), a time when locomotor activity peaks and the probability of swarming increases (figure 1*a;* [25,36]). We identified 27 differentially expressed transcripts (*p* < 0.05, log_2_FC > |0.75|); 11 were upregulated and 16 downregulated in swarming individuals (figure 1*b* and table 1). Contrary to previous findings in *Anopheles coluzzii* (part of the *gambiae* complex) [20], the clock genes *period (per*) and *timeless (tim*) were not upregulated in swarming males (figure 1*b*). Differentially expressed genes were annotated using VectorBase [28]. The annotation classified 17 of the 27 transcripts as ‘unspecified products’, of which four (one upregulated and three downregulated) were reported lacking orthologues in *D. melanogaster*. However, using OrthoDB [34], we identified three of them (table 1). We used the fly orthologues and FlyBase to infer gene function [35].

**Table 1.** List of genes upregulated and downregulated in swarming *Aedes aegypti* males. *Drosophila* orthologues and their function are also reported. Asterisk indicates orthologues identified using OrthoDB [34].

	VectorBase ID	LogFC	VectorBase description	fly gene name	fly gene symbol
upregulated transcripts	AAEL025907-RA	8.81120219008974	unspecified product	*Pyruvate dehydrogenase phosphatase*	*Pdp*
	AAEL010711-RD	8.05491679463046	eph receptor tyrosine kinase	*Eph receptor tyrosine kinase*	*Eph*
	AAEL008603-RH	7.6970423099866	unspecified product	FBtr0346461/FBtr0079092/FBtr0342941/FBtr0342940	*CG7742*
	AAEL009895-RI	6.98034185062882	neprilysin	*Neprilysin*	*Nep*
	AAEL009211-RC	6.89770142690902	unspecified product	*Bardet–Biedl syndrome 1*	*BBS1*
	AAEL000181-RF	6.85649475915555	polybromo−1	*polybromo*	*polybromo*
	AAEL027977-RB	6.07788099164431	unspecified product	*lethal (3) neo43*	*l(3)neo43*
	AAEL019804-RF	5.26486228341179	unspecified product	*5-hydroxytryptamine (serotonin) receptor 2A*	*5-HT2A*
	AAEL018306-RF	2.01910788720389	unspecified product	*slowpoke*	*slo*
	AAEL022180-RB	1.28374810701443	unspecified product	*Cdc42-interacting protein 4*	*Cip4*
	AAEL027189-RA	1.23244554902235	unspecified product	no orthologues	n.a.
	AAEL003228-RB	0.964354629261942	mitotic protein phosphatase 1 regulator	*flyers-cup*	*f-cup*
downregulated transcripts	AAEL020244-RA	−9.90706779481855	serine/threonine-protein phosphatase 2A activator	*Phosphotyrosyl phosphatase activator*	*Ptpa*
	AAEL025228-RD	−9.02913486903167	unspecified product	no orthologues	n.a.
	AAEL027400-RG	−8.98939278971224	unspecified product	FBtr0331339/FBtr0331340/FBtr0113327/FBtr0070181	*CG3655*
	AAEL020264-RD	−8.77196159862634	unspecified product	*sidekick*	*sdk*
	AAEL005950-RS	−7.3053249062435	chloride channel protein 2	*Chloride channel-a*	*ClC-a*
	AAEL002879-RS	−7.04851442679623	heterogeneous nuclear ribonucleoprotein r	*Syncrip*	*Syp*
	AAEL025228-RC	−6.39078895206306	unspecified product	chiffon*	chif*
	AAEL021054-RA	−5.54499930568334	unspecified product	*Inositol polyphosphate−5-phosphatase type I*	*5Ptasel*
	AAEL004731-RE	−1.51420598905933	unspecified product	*Zizimin-related*	*Zir*
	AAEL027790-RF	−1.399664586898	unspecified product	Tenectin isoform 1*	Tnc*
	AAEL018348-RD	−1.23873378750299	unspecified product	*Autophagy-related 17*	*Atg17*
	AAEL019711-RC	−1.17663412042304	unspecified product	*Phosphoinositide phospholipase C*	*Plc21C*
	AAEL006283-RA	−1.14112366907515	GPCR myosuppressin	*Myosuppressin receptor*	*MsR*
	AAEL022806-RC	−1.12954115169289	unspecified product	FBtr0340116/FBtr0346189/FBtr0074104	*CG8578*
	AAEL019898-RF	−0.86508645496451	unspecified product	uncharacterized protein*	CG30015*

In swarming males, upregulated genes included *Pyruvate dehydrogenase phosphatase* (*Pdp*, AAEL025907-RA) that is involved in both metabolic and signalling pathways, *Ephrin tyrosin kinase receptor* (*Eph*, AAEL010711-RD) whose product promotes cell adhesion, axon guidance and the maintenance of sleep and circadian rhythms [23,37], *polybromo* (AAEL000181-RF) encoding a chromatin remodeller, and *serotonin receptor 2A* (*5-*HT2A, AAEL019804-RF) implicated in food choice, ageing and modulation of circadian behaviour [22,38]. Additional genes of interest were *slowpoke* (*slo*, AAEL018306-RF) whose product is a BK channel involved in odour-recognition in *A. gambiae* [39] and in the synchronization of circadian neurons in flies [21]; and *Bardet–Bield syndrome 1* (*BBS1*, AAEL009211-RC) that encodes a protein expressed in sensory neurons in *Drosophila*, important for cilia biogenesis [40]. Indeed, gene ontology analyses revealed an enrichment in the categories: cell communication, ephrin receptor signal pathway and cilium assembly (tables 1 and 2).

**Table 2.** List of Gene Ontology (GO) terms that are enriched in relation to upregulated and downregulated transcripts in swarming *Ae. aegypti* males. GO terms for molecular function (MF) and biological process (BP) domains are shown. The statistical significance of the enrichment is reported in the *P*_adj_ column. Results are from g:Profiler [33].

	GO domain	GO term ID	GO term name	*P*_adj_
upregulated transcripts	BP	GO:0048013	ephrin receptor signalling pathway	1.05 × 10^−2^
	BP	GO:0006793	phosphorus metabolic process	2.81 × 10^−2^
	BP	GO:0006796	phosphate-containing compound metabolic process	2.81 × 10^−2^
	BP	GO:0007169	transmembrane receptor protein kinase signalling pathway	2.81 × 10^−2^
	BP	GO:0036211	protein modification process	2.81 × 10^−2^
	BP	GO:19 05 515	non-motile cilium assembly	2.81 × 10^−2^
	BP	GO:0006470	protein dephosphorylation	2.86 × 10^−2^
	BP	GO:0007167	enzyme-linked receptor protein signalling pathway	2.86 × 10^−2^
	BP	GO:0043412	macromolecule modification	2.86 × 10^−2^
	BP	GO:0016311	dephosphorylation	3.31 × 10^−2^
	BP	GO:0019538	protein metabolic process	4.62 × 10^−2^
	MF	GO:0005003	ephrin receptor activity	9.92 × 10^−3^
	MF	GO:0060072	large conductance calcium-activated potassium channel activity	2.23 × 10^−2^
	MF	GO:0004714	transmembrane receptor protein tyrosine kinase activity	2.48 × 10^−2^
	MF	GO:0004722	protein serine/threonine phosphatase activity	2.48 × 10^−2^
	MF	GO:0005227	calcium-activated cation channel activity	2.48 × 10^−2^
	MF	GO:0015269	calcium-activated potassium channel activity	2.48 × 10^−2^
	MF	GO:0019199	transmembrane receptor protein kinase activity	2.48 × 10^−2^
	MF	GO:0140096	catalytic activity, acting on a protein	2.48 × 10^−2^
	MF	GO:0004721	phosphoprotein phosphatase activity	3.24 × 10^−2^
	MF	GO:0004713	protein tyrosine kinase activity	3.24 × 10^−2^
	MF	GO:0003682	chromatin binding	3.4 × 10^−2^
downregulated transcripts	BP	GO:0000422	autophagy of mitochondrion	2.50 × 10^−2^
	BP	GO:19 03 008	organelle disassembly	2.50 × 10^−2^
	BP	GO:0061726	obsolete mitochondrion disassembly	2.50 × 10^−2^
	MF	GO:0052743	inositol tetrakisphosphate phosphatase activity	5.87 × 10^−3^
	MF	GO:0004445	inositol-polyphosphate 5-phosphatase activity	5.87 × 10^−3^
	MF	GO:0052659	inositol-1,3,4,5-tetrakisphosphate 5-phosphatase activity	5.87 × 10^−3^
	MF	GO:0052658	inositol-1,4,5-trisphosphate 5-phosphatase activity	5.87 × 10^−3^
	MF	GO:0046030	inositol trisphosphate phosphatase activity	5.87 × 10^−3^
	MF	GO:0019211	phosphatase activator activity	5.87 × 10^−3^
	MF	GO:0019208	phosphatase regulator activity	9.34 × 10^−3^
	MF	GO:0005247	voltage-gated chloride channel activity	1.26 × 10^−2^
	MF	GO:0003755	peptidyl-prolyl *cis*–*trans* isomerase activity	1.37 × 10^−2^
	MF	GO:0016859	*cis*–*trans* isomerase activity	1.37 × 10^−2^
	MF	GO:0008308	voltage-gated monoatomic anion channel activity	1.37 × 10^−2^
	MF	GO:0052745	inositol phosphate phosphatase activity	1.58 × 10^−2^
	MF	GO:0004435	phosphatidylinositol phospholipase C activity	1.58 × 10^−2^
	MF	GO:0004629	phospholipase C activity	1.58 × 10^−2^
	MF	GO:0008047	enzyme activator activity	2.01 × 10^−2^
	MF	GO:0042578	phosphoric ester hydrolase activity	2.28× 10^−2^
	MF	GO:0016853	isomerase activity	2.28 × 10^−2^
	MF	GO:0005254	chloride channel activity	2.28 × 10^−2^
	MF	GO:0030234	enzyme regulator activity	2.28 × 10^−2^
	MF	GO:0005253	monoatomic anion channel activity	2.38 × 10^−2^
	MF	GO:0015108	chloride transmembrane transporter activity	2.62 × 10^−2^
	MF	GO:0140677	molecular function activator activity	2.62 × 10^−2^
	MF	GO:0098772	molecular function regulator activity	2.94 × 10^−2^
	MF	GO:0015103	inorganic anion transmembrane transporter activity	3.03 × 10^−2^
	MF	GO:0022832	voltage-gated channel activity	3.37 × 10^−2^
	MF	GO:0005244	voltage-gated monoatomic ion channel activity	3.37 × 10^−2^
	MF	GO:0008509	monoatomic anion transmembrane transporter activity	3.61 × 10^−2^

Among the downregulated genes, a common theme was involvement in housekeeping functions. *Phosphotyrosyl phosphatase activator* (*Ptpa*, AAEL020244-RA) is implicated in cell fate determination, mitotic spindle organization and basal protein localization. The gene *sidekick* (*sdk*, AAEL020264-RD) regulates remodelling of epithelia and synapses. *Syncrip* (*Syp*, AAEL002879-RS) encodes an RNA-binding protein involved in many biological processes including the negative regulation of synaptic assembly. The product of *chiffon* (*chif*, AAEL025228-RC) mediates cell division and cell cycle progression. Additional biological processes that may be affected are immune responses (*Zir*, AAEL004731-RE), autophagy (*Atg17*, AAEL018348-RD), contractility of visceral muscles (*MsR1*, AAEL006283-RA) and inositol metabolism (*5Ptasel*, AAEL021054-RA), which is important for energy homeostasis [41]. Accordingly, organelle disassembly, autophagy of mitochondrion and inositol triphosphate phosphatase activity were enriched ontology terms (tables 1 and 2).

These modifications in gene expression may prepare the physiological changes in flight speed and in acoustic perception that are observed in swarming males in several species of mosquitoes [42,43].

## 4. Discussion

Swarming is fundamental for the biology of mosquitoes. It marks the time and place where males aggregate to attract females and compete for mating. It is a challenging behaviour that involves several sensory modalities and rapid sensory–motor integration; among many males only the fastest in recognizing and catching a female in mid-flight will be able to mate. This study was aimed at identifying gene expression changes that accompany swarming. Our findings indicate that males prepare for such a behaviour upregulating genes involved in cytoskeletal reorganization and heightened cellular connectivity, while decreasing the expression of genes regulating various housekeeping functions. These changes are likely facilitated by the activation of chromatin remodelling pathways (figure 1*b* and table 1). It is revealing that swarming in *Ae. aegypti* may require promoting processes expected to redefine cellular performance and connectivity, and we speculate that they contribute to ‘upscaling’ motor abilities and sensory acuity. Physiological and structural changes in connection with swarming have been documented for flying and hearing [42–44]. These may also occur for other sensory modalities such as vision or detection of infrared radiance [45,46].

Recent work carried out in the malaria mosquito *A. coluzzii* identified increased expression of the canonical clock genes *per* and *tim* in swarming males in the field [20]. Additionally, the authors described increased expression for carbohydrate, amino acid or lipid metabolism genes and an overall reduction for cytoskeletal and structural genes [20]. In *Ae. aegypti*, we did not detect differential expression for clock genes (figure 1*b*), and the expression of metabolic and cytoskeletal genes showed an opposite trend (table 1). Furthermore, we observed no overlap in differentially expressed genes between the two species. Wang and co-workers [20] collected *A. coluzzii* mosquitoes at dusk from the wild, sampling males that were flying outdoors in swarms and males that were at rest in houses, two very different environments. We sampled *Ae. aegypti* in the laboratory, collecting swarming and non-swarming males from the same cage. Our experimental design has the advantage of comparing two different behavioural states, that we defined as swarming and non-swarming, under the same environmental conditions. However, one obvious limitation is that in the laboratory it is difficult to distinguish swarming, defined as sustained aggregated flight, from other behaviours (for instance, arousal) that involve flying. Thus, the disparity in the list of genes showing differential expression between *Anopheles* and *Aedes* may reflect to some extent the different experimental conditions. However, we suggest that species-specific differences also contribute. Indeed, in the nocturnal *Anopheles* species*,* swarming is strictly crepuscular in the wild and tightly timed by the circadian clock, as shown in laboratory settings [9,42,47–49]. Conversely, in the diurnal *Ae. aegypti*, the timing of swarming is less restricted, although the clock still maintains some control, as the behaviour occurs mainly towards the end of the day, which coincides with the peak in flight activity at ZT10.5–11 [16,25]. Thus, future work should consider comparing swarming and non-swarming in the laboratory and in the wild for both species.

An important question concerning the biology of mosquitoes is whether the clock is generally (i.e. in most species) involved in swarming, either *per se* or through pleiotropic effects. Indeed, *period* and other clock genes affect courtship, reproduction and innate routine behaviours in several species, from insects to vertebrates [50–56]. Then, it may not be surprising that both *Anopheles* and *Aedes* show a reduction in mating and fertility when the clock is disrupted, confirming that the clock, or its components, affects processes in addition to biological rhythmicity also in mosquitoes.

Even though canonical clock genes were not differentially expressed in *Ae. aegypti*, it is notable that the gene *slowpoke* (*slo*) was upregulated in swarming males. This gene encodes a calcium-dependent potassium channel that regulates circadian locomotor activity in *D. melanogaster* [21,57,58]. Indeed, several groups of (dorsal) circadian neurons are desynchronized in *slo*-null flies due to a reduction in the release of the neuropeptide pigment dispersing factor (PDF). PDF is produced by an important cluster of clock neurons and functions as a synchronization agent across the neuronal clock network [58]. Moreover, in flies, SLO is directly involved in acquiring circadian information in neuronal circuits downstream of the clock that are necessary for the maintenance of locomotor activity rhythms [57]. Interestingly, *slo* mutations cause changes to the courtship song in *Drosophila*, a sound generated by males vibrating their wings [59]. As with flies, the organization of the circadian network in *A. coluzzii* and in *Ae. aegypti* is based on the expression of PER and PDF, although species-specific differences exist [60]. A further similarity lies in the fact that although they do not generate a song, males of many mosquito species change the frequency of their wingbeat for mating [18,19,42]. Thus, it is tempting to speculate that *slo*, like in *Drosophila*, may have circadian and ‘mating’ roles in *Aedes*. In summary, our work suggests that in mosquitoes, the circadian neuronal network supports swarming beyond the direct involvement of the molecular cogs of the clock. Testing this hypothesis will motivate our future efforts.

## Data Availability

Data have been deposited in GenBank under NCBI BioProject: PRJNA1148942. All codes are available from the Zenodo Repository: https://doi.org/10.5281/zenodo.13330053.
